# Practice of ST-segment elevation myocardial infarction care in the Netherlands during four snapshot weeks with the National Cardiovascular Database Registry for Acute Coronary Syndrome

**DOI:** 10.1007/s12471-017-0947-6

**Published:** 2017-01-31

**Authors:** N. P. G. Hoedemaker, M. E. ten Haaf, J. C. Maas, P. Damman, Y. Appelman, J. G. P. Tijssen, R. J. de Winter, A. W. J. van ‘t Hof

**Affiliations:** 1 Department of Cardiology, Academic Medical Centre, University of Amsterdam, Amsterdam, The Netherlands; 2 Department of Cardiology, VU Medical Centre, VU University Amsterdam, Amsterdam, The Netherlands; 3 National Cardiovascular Data Registry, Utrecht, The Netherlands; 4 Isala Klinieken Hospital, Zwolle, The Netherlands

**Keywords:** ST-elevation myocardial infarction, Registry, Acute coronary syndrome

## Abstract

**Background:**

Clinical registries provide information on the process of care and patient outcomes, with the potential to improve the quality of patient care. A large Dutch national acute coronary syndrome (ACS) registry is currently lacking. Recently, we initiated the National Cardiovascular Database Registry (NCDR) for ACS in the Netherlands. The purpose of this study was to assess the NCDR ACS registry on feasibility and data completeness during a pilot phase of four snapshot weeks.

**Methods:**

Between 2013 and 2015, we invited all hospitals in the Netherlands to record a predefined dataset for every patient that was admitted to their hospital with ST-segment elevation myocardial infarction (STEMI). Data were entered in an online case report form. All patient-specific data were encrypted to ensure privacy.

**Results:**

A total of 392 patients were registered in 35 centres. The mean age of the patients was 64 years (SD 13); 8% of patients presented with signs of cardiogenic shock and 11% with an out-of-hospital cardiac arrest. The median time from first medical contact to percutaneous coronary intervention (PCI) was 75 min (IQR 51–108) and this was significantly longer for patients who presented at a non-PCI centre or to a primary care physician. In-hospital and 30-day mortality rates were 5.2% and 7.8%, respectively. The amount of completeness varied, with improved completeness over time.

**Conclusion:**

This report shows that a Dutch ACS registry is feasible with respect to STEMI patients. Data completeness, however, was suboptimal. Improved data completeness is warranted for the future.

## Introduction

Within the spectrum of coronary artery disease, acute coronary syndrome (ACS) is a major cause of death, hospitalisation, and high rates of acute complications. In the past decades, there have been improvements in the management of ACS patients. This resulted in a decrease in mortality and morbidity of ACS patients, as shown by registry data [1, 2]. In addition to monitoring outcomes and quality of care, registries provide the opportunity for hospitals to evaluate the implementation of clinical guidelines. Furthermore, benchmarking of hospital performance has the potential to ultimately improve the quality of care for ACS patients. Finally, national registries provide information on ACS patients who are underrepresented in randomised controlled trials (RCT), for example the elderly and shock patients.

Successful national registration of ACS patients is performed in Sweden, the United States and the United Kingdom and more recently in France, Germany, Poland, and Switzerland [3–9]. In the Netherlands, the National Cardiovascular Data Registry (NCDR) aims to collect national data on cardiovascular interventions and device implantations (http://www.ncdr.nl). In addition to the existing registries, the NCDR ACS registry was recently initiated. Hospitals that provide relevant services participate in the collection of data on ST-segment elevation myocardial infarction (STEMI) patients. We report the results of the first four snapshot test weeks of this registry, aiming to assess the feasibility and data completeness of a Dutch ACS registry.

## Methods

### Source data and study population

We used data that derived from the NCDR ACS registry. This registry has enrolled consecutive ACS patients admitted to hospitals in the Netherlands since September 2013. Information is collected prospectively, and the variables in the registry comply with the International Cardiology Audit and Registration Data Standards (CARDS). A description in Dutch is available at http://www.ncdr.nl/registraties/acs. The current analysis consists of all patients presenting with a registered admission diagnosis of STEMI during the four snapshot test weeks held in September 2013, March and September 2014, and May 2015. Hospitals that participated in the data collection are listed in the Appendix. The NCDR ACS working group approved the registry and the current analysis. According to Dutch law, no written informed consent was required.

### Organisation and funding

NCDR is an independent organisation that was founded by the Netherlands Society of Cardiology. The NCDR provides multiple clinical registries, including the ACS registry. This registry is managed by a steering group which includes members of the ACS working committee of the Netherlands Society of Cardiology. The steering group takes care of project management, monitoring, quality and statistical reports. The NCDR ACS registry is self-funded by the participating hospitals and independent from commercial funding. The participating hospitals retain ownership of the patient data they provide.

### Data management

Data are stored on an externally located server and managed by NCDR and Reports BV (Almere, the Netherlands). NCDR is NEN 7510:2011 (a Dutch institute supporting standardisation for data protection) certified for information security in Dutch healthcare. A trusted third party (Zorg TTP, Houten, the Netherlands) encrypts all confidential patient information before it is stored. Independent researchers do not have access to the decryption key. The NCDR ACS dataset includes patient demographics and risk factors, time to treatment, details of reperfusion treatment, and outcomes up to 30 days. The NCDR ACS registry has automatic error-checking routines, including range and consistency checks. For this paper, data were exported from the database on 24 August 2015.

### Definitions

First medical contact (FMC) was defined as the first patient contact with a medical care giver. FMC was subdivided into 1) ambulance (emergency service call), 2) emergency room at a centre performing percutaneous coronary interventions (PCI), 3) emergency room at a non-PCI centre and 4) primary care physician. FMC time was defined as the time of the first diagnostic ECG. PCI time was defined as the time of arrival at the PCI centre. Total ischaemic time was defined as the time from symptom onset until arrival at the PCI centre. Cardiogenic shock was defined as signs of shock (systolic blood pressure <90 mm Hg, tachycardia, decreased peripheral circulation, cold extremities, and Killip class 3 or 4) during admission at the PCI centre. Out-of-hospital-cardiac arrest (OHCA) was defined as cardiac resuscitation initiated before admission to the hospital.

### Statistical analysis

Continuous variables are presented as the mean with standard deviation or median with interquartile range depending on their distribution. Categorical variables are presented as the number with percentage. Comparison of time between different first FMC categories and time of PCI was performed with the Kruskal-Wallis test and Dunn’s multiple comparison test. *P*-values <0.05 were considered significant.

## Results

### Baseline characteristics, time delay, and treatment strategies

The baseline characteristics and treatment strategies are presented in Table 1. In September 2013, March and September 2014, and May 2015, a total of 329 patients with a STEMI were included in 35 centres. The mean age was 64 years (SD 13) and approximately one-third of the patients were women. At the time of admission, half of the patients were smokers. Of the patients, 8% presented with signs of cardiogenic shock and 11% with an OHCA.

**Table 1:** Baseline characteristics of the patients and treatments

	Week 1		Week 2		Week 3		Week 4		Combined
STEMI patients	*N* = 120	Na/*N*	*N* = 123	Na/*N*	*N* = 78	Na/*N*	*N* = 71	Na/*N*	*n* = 392
*Patient characteristics*									
Mean age (SD)	65 (12)	0/120	64 (14)	0/123	61 (13)	0/78	64 (13)	0/71	64 (13)
Women	34.2%	0/120	32.5%	0/123	29.5%	0/78	21.1%	0/71	30.4%
Current smokers	60.3%	57/120	36.5%	38/123	48.3%	20/78	53.7%	17/71	48.5%
Cardiogenic shock	9.6%	16/120	8.8%	32/123	9.2%	13/78	4.5%	4/71	8.3%
OHCA	5.7%	15/120	12.2%	33/123	16.9%	13/78	10.1%	2/71	10.6%
*First medical contact*									
Ambulance	64.1%	17/120	65.5%	7/123	63.8%	9/78	62.8%	1/71	64.2%
ER PCI centre	13.6%	17/120	6.0%	7/123	5.8%	9/78	4.3%	1/71	7.8%
ER non-PCI centre	9.7%	17/120	21.6%	7/123	15.9%	9/78	18.6%	1/71	16.5%
Primary care physician	12.6%	17/120	6.9%	7/123	14.5%	9/78	14.3%	1/71	11.5%
*Intervention*									
CAG	100%	0/120	100%	0/123	100%	8/78	86.8%	3/71	95.6%
Radial access	45.9%	11/120	54.2%	16/123	71.6%	3/70	86.2%	1/59	60.4%
PCI after CAG	95.6%	75/120	94.4%	15/123	94.0%	3/70	96.6%	0/59	95.0%
Thrombus aspiration	69.2%	30/43	55.0%	82/102	34.6%	11/63	28.90%	19/57	39.8%
Successful PCI	100%	25/43	100%	57/102	98.3%	5/63	97.5%	17/57	98.8%
*Time delay*									
FMC-to-PCI time	75 (48–152)	16/43	80 (52–109)	20/102	74 (57–112)	13/63	61 (51–92)	14/57	75 (51–108)
Total ischaemic time	163 (75–440)	18/43	165 (126–246)	23/102	167 (102–328)	15/63	168 (107–293)	14/57	165 (106–287)

*Na* number of missing values filled in on the case report form, *N* number of completed case report forms available, *STEMI* ST-segment elevation myocardial infarction, *SD* standard deviation, *OHCA* out-of-hospital cardiac arrest, *ER* emergency room, *PCI* percutaneous coronary intervention, *IQR* interquartile range, *FMC* first medical contact, *CAG* coronary angiography

Time is in minutes

The median FMC-to-PCI time was 75 min (IQR 51–108). A total of 66.2% of the patients first presented in an ambulance and these patients had the lowest FMC-to-PCI time (69 min, IQR 50–92). Patients presenting in an ambulance also had a significantly lower FMC-to-PCI time compared with those who presented at a non-PCI centre or a primary care physician, as is displayed in Table 2.

**Table 2:** Time of first medical contact to percutaneous coronary intervention

First medical contact	*N*	Percentage	Time to PCI (min)	*p*-value*
Ambulance	133	66.2	69 (50–92)	–
ER PCI centre	14	7.0	78.5 (46.5–126.3)	NS
ER non-PCI centre	34	16.9	97.5 (64.5–129)	*p* < 0.05
Primary care physician	20	9.9	96 (65.8–174.3)	*p* < 0.01

*ER* emergency room, *PCI* percutaneous coronary intervention, *NS* not significant, * compared with ambulance

Time displayed as mean with interquartile range

Catheterisation was performed via the radial artery in 60.4% of the patients and thrombus aspiration was used in 39.8%. There was an increase in the use of radial access and a decrease in the use thrombus aspiration in 2015, compared with 2013. Primary PCI was performed in 95.0% of patients, of which 98.8% was successful.

### Outcomes

The rate of in-hospital complications was 13.5%, as is displayed in Table 3. The rates of in-hospital and 30-day mortality were 5.2% and 7.8% respectively. The use of dual antiplatelet therapy, beta-blockers and statins was slightly under 90% and anticoagulants were prescribed in 18.5%. Of the patients, 87.7% were offered a place in a cardiac rehabilitation program of whom 80.9% are known to have participated.

**Table 3:** Outcomes, medication at discharge and cardiac rehabilitation

	Week 1		Week 2		Week 3		Week 4		Combined
STEMI patients	*N* = 120	Na/*N*	*N* = 123	Na/*N*	*N* = 78	Na/*N*	*N* = 71	Na/*N*	*N* = 392
*Outcomes*									
In-hospital complications	14.5%	51/120	7.3%	41/123	28.6%	50/78	14.0%	21/71	13.5%
In-hospital mortality	2.9%	51/120	2.4%	41/123	14.30%	50/78	8.0%	21/71	5.2%
30-day mortality	3.5%	35/120	7.1%	67/123	13.2%	40/78	13.2%	33/71	7.8%
*Medication at discharge*									
Acetylsalicylic acid	82.5%	23/120	90.6%	38/123	95.8%	30/78	93.3%	41/71	88.8%
P2Y12 inhibitors	82.8%	21/120	91.7%	27/123	93.90%	29/78	80.0%	41/71	87.6%
Beta-blocker	67.4%	25/120	81.3%	43/123	93.0%	35/78	93.3%	41/71	87.6%
Statin	80.4%	28/120	91.4%	42/123	95.3%	35/78	93.3%	41/71	88.2%
Anticoagulants	15.7%	31/120	25.4%	52/123	50.0%	39/78	3.6%	43/71	18.5%
*Cardiac rehabilitation*									
Cardiac rehabilitation proposed	91.8%	59/120	88.2%	72/123	78.8%	45/78	88.0%	46/71	87.7%
Cardiac rehabilitation attended	70.0%	16/56	91.7%	21/45	88.2%	9/26	77.8%	13/22	80.9%

*Na* number of missing values filled in on the case report form, *N *number of completed case report forms available

*STEMI* ST-segment elevation myocardial infarction

### Missing data

Information on missing data can be found in Table 1 and 3. The data completeness of patient characteristics and information on FMC and intervention improved over the course of the four weeks. Data completeness for these categories generally increased by 50% or more. The percentage of missing data on time delay indicators decreased slightly.

The amount of missing data on in-hospital outcome was lower in 2015 compared with 2013, whereas missing data on 30-day mortality increased. In addition, an increase in missing data was also found for information on discharge medication and cardiac rehabilitation.

## Discussion

The current report describes the first results of the Dutch NCDR ACS registry with respect to STEMI patients. The first data were collected during four snapshot test weeks between 2013 and 2015 and show that a national registry for STEMI patients is feasible. However, with suboptimal data completeness, improvement is of upmost importance.

### Outcomes of an unselected population

This registry showed in-hospital and 30-day mortality rates of 5.2% and 7.8%, respectively. To put this into context, 30-day mortality rates of STEMI patients enrolled in various major RCTs conducted from 2009 to 2013 ranged from 2.0–3.3% [10–13]. For this registry no patients were excluded and we aimed to collect data that reflect real-world practice. Almost 10% of patients presented after an OHCA and 8% presented in cardiogenic shock. In addition, 5% of patients did not receive reperfusion therapy, mainly because of late presentation or frailty. This may explain the higher mortality rate. A higher in-hospital mortality rate was also found in Denmark (10.9%, 95% CI 7.0–14.7) and in a snapshot of Western European countries (6.3%) [14, 15]. In addition, the Swedish and the British registries show 30-day mortality rates of 8.6% (95% CI 8.3–8.8) and 11.2% (95% CI 11.1–11.4) respectively [1]. The results from our registry showed lower mortality rates; however, these derived from a small sample size and thus, comparison with results from other registries should be done with care.

### Trends in practice

Registries can help to identify trends in practice of care over the years. Our results show an increase in the use of radial access and a decrease in the use of thrombus aspiration in 2015, compared with the first snapshot test week in 2013. These results may reflect the impact of the latest trials that were conducted in these areas and follow recommendations of guidelines [16–20].

### National registries

Clinical registries are necessary in order to monitor outcomes and ultimately help improve the quality of care. Examples of successful national registries include the Myocardial Ischaemia National Audit Project (MINAP) from the United Kingdom, the American NCDR CathPCI registry, and the Swedish Web-system for Enhancement and Development of Evidence-based care in Heart disease Evaluated According to Recommended Therapies (SWEDEHEART) [7–9]. The SWEDEHEART registry has demonstrated the positive impact of evidence-based treatments on outcomes. In a report that included STEMI patients between 1996–2007, an increase in the use of evidence-based treatments was associated with a sustained decrease in 30-day and 1‑year mortality during the same period of time [21]. In addition, registries have expanded their roles and are also used for multi-centre observational research and more recently, registry-based RCTs [22]. Furthermore, international collaborations can be initiated, potentially giving insight into differences in patient characteristics and treatments among countries [23, 24].

At a national level, the NCDR ACS registry can be used as an instrument for quality improvements in hospitals. A retrospective study showed that Dutch hospitals use dissimilar definitions to determine performance indicators for STEMI patients [25]. The use of predefined key performance and quality indicators, which correspond with those required by the Dutch Health Inspectorate and the Dutch Safety Management System, can solve this problem and offers a universal method to compare hospitals at a national level. This can potentially create opportunities to reduce complications, and reveal targets for improvement.

### Future perspectives

This report shows the first test on feasibility and data completeness of a national Dutch ACS registry. A total of 35 centres have participated at least once, and have thereby been introduced to be part of the NCDR ACS registry and the process of providing data to NCDR. In order to catch up with registries from surrounding countries, we need to further develop the Dutch registration.

Firstly, our main goal is to involve all Dutch hospitals in the registry and start continuous inclusion of all ACS patients. However, a common problem for hospitals is the workload of the registration, which is needed to guarantee the quality and completeness of the data. Currently, not every Dutch hospital has a registration nurse, which may cause hospitals to provide an incomplete dataset or to withdraw from the national registry. Therefore, we encourage all Dutch hospitals to recruit a dedicated registration nurse. Possibly, this could be supported by Dutch government institutions or health insurance companies, since a successful registry can contribute to improving the quality of care. In addition, we want to develop ways to link the national registry to insurance, pharmacy, and hospital databases, in order to collect follow-up data beyond 30 days after admission.

Secondly, we aim to expand the ACS registry to include patients with non-ST-elevation-ACS (NSTE-ACS). Registration of NSTE-ACS can be challenging because of the heterogeneity of the population; however, it will provide further insight into the use of antiplatelet and antithrombotic therapy or differences between PCI and non-PCI centres regarding timing of intervention in NSTE-ACS [26–28].

For the future, we want to integrate and link different electronic patient records (EPRs) in order to allow direct data capture from the patient records, which will minimise the extra work associated with data entry. The first experiences with direct registration from existing EPRs are encouraging. As mentioned previously, the NCDR ACS registry will serve as a platform for national quality improvement efforts. Beyond this primary goal, international collaborations and research will be important other goals.

### Limitations

Several limitations must be taken into account. Our results are based on registry data and may be subject to some selection bias. We invited all hospitals in the Netherlands to report consecutive STEMI cases; however, participation in the registry was voluntary. Overall, 18 of 55 non-PCI (32%) and 17 of the 30 PCI (57%) centres participated and therefore our results may not fully reflect the whole STEMI population in the Netherlands. Despite this limited participation and patient numbers, our results seem to provide an adequate representation of an unselected Dutch STEMI population.

## Conclusion

The NCDR ACS registry is a national program that aims to enrol consecutive ACS patients presenting to hospitals in the Netherlands. The results from four snapshot test weeks show that a Dutch national ACS registry is feasible. Data completeness, however, was suboptimal. Improved data completeness, is warranted for the future.

## Appendix

### NVVC ACS working group

A.W.J. van ’t Hof (chair), Y. Appelman, J.M. ten Berg, A.H. Liem, W.R.P. Agema, T.J.F. ten Cate, P. Damman, I.J van Eede, D.J. van der Heijden, J.W. Jukema, G.J. Laarman, R.J.G. Peters, M.L.J. Schaik-van der Wielen, P.W.J.C. Serruys, W.A.L. Tonino, R.A. Waalewijn, F.F. Willems, R.J. de Winter

### NCDR ACS steering committee

A.W.J. van ’t Hof (chair), J.M. ten Berg, P. Damman, J.G.P.Tijssen, V.A.W.M. Umans, T.W. Galema

### NCDR PCI steering committee

W.R.M. Aengevaeren, Y. Appelman, A.W.J. van ’t Hof, M. Meuwissen, A.A.C.M. Heestermans, J.G.P. Tijssen, VA.W.M. Umans, T.W. Galema

### List of participating centres

Academic Medical Center – University of Amsterdam, Albert Schweitzer Hospital, Alrijne Hospital Leiderdorp, Amphia Hospital, Antonius Hospital, Bethesda Hospital, BovenIJ Hospital, Bronovo Hospital, Catharina Hospital, Deventer Hospital, Sint Franciscus Gasthuis Hospital, Gelre Hospital Apeldoorn, Gemini Hospital, Groene Hart Hospital, Haga Hospital Leyweg, Isala Hospital, Jeroen Bosch Hospital , Leiden University Medical Center, Maasstad Hospital, Medical Center Alkmaar, Rijnstate Hospital, Rode Kruis Hospital, St. Antonius Hospital, TweeSteden Hospital, University Medical Center Groningen, Münster University Hospital, University Medical Center St Radboud, VU Medical Center Westfries Gasthuis Hospital, Hospital Rivierenland, Hospital St Jansdal, Ziekenhuisgroep Twente Hospital (Hengelo), Zorggroep Noorderbreedte Hospital, ZorgSaam Zeeuws-Vlaanderen Hospital, Zuyderland Medical Center Heerlen
